# Impact of Adjuvant Cemiplimab in High-Risk Cutaneous Squamous Cell Carcinoma

**DOI:** 10.3390/curroncol32080459

**Published:** 2025-08-15

**Authors:** Annette M. Lim, Sandro Porceddu, Danny Rischin

**Affiliations:** 1 Department of Medical Oncology, Peter MacCallum Cancer Centre, 3000 Melbourne, Australia; annette.lim@petermac.org (A.M.L.); sandro.porceddu@petermac.org (S.P.); 2 Sir Peter MacCallum Department of Oncology, University of Melbourne, 3000 Melbourne, Australia; 3 Department of Radiation Oncology, Peter MacCallum Cancer Centre, 3000 Melbourne, Australia

Despite cutaneous squamous cell carcinoma (CSCC) being the second most common skin cancer worldwide, there were no approved systemic therapies for patients with unresectable and/or metastatic disease prior to the advent of anti-programmed cell death protein-1 (anti-PD1) agents cemiplimab and pembrolizumab [1,2]. The paradigm of CSCC management has been recently revolutionised with the landmark success of the cPOST trial, which investigated the efficacy of adjuvant cemiplimab versus placebo in patients with high-risk CSCC following surgery and radiation therapy [3]. After median follow-up of 24 months, cemiplimab achieved a striking improvement in disease-free survival (DFS) with a hazard ratio (HR) of 0.32 (95% confidence interval [CI], 0.20 to 0.51; *p* < 0.001) and no adverse impacts on patient-reported quality of life (QOL) compared to placebo. The cPOST study represents the first positive randomised phase 3 trial ever conducted in CSCC. However, as one considers implementation, the success of the cPOST trial also highlights differences and controversies in global practice warranting further research and harmonisation efforts.

One of the key achievements of the cPOST trial is in the robust validation of a clinicopathological definition of “high-risk” CSCC to appropriately select patients who will benefit from adjuvant cemiplimab. This is juxtaposed against the lack of international consensus regarding the optimal staging systems of CSCC, which differ in terms of variables used to risk-stratify, contain criteria without international consensus such as differentiation status, differ in their ability to predict recurrence risk versus overall survival, differ in terms of their context for use (e.g., all-comers versus immunocompromised patients, advanced versus early-stage disease), and differ in risk categories (e.g., low/high or low/intermediate/high risk categories) [4,5,6,7,8,9,10,11]. Critically, the staging systems do not directly inform treatment decisions for patients, particularly those with locoregionally advanced CSCC. For example, the Brigham Women’s Hospital (BWH) criteria are primarily useful for prognostication in lower staged and lower risk CSCC, which is irrelevant to the cPOST-eligible trial population [4,11,12]. Furthermore, the threshold percentage risk of CSCC recurrence derived from the staging systems that warrants adjuvant therapy is contentious, with an arbitrary 20% threshold of risk used to guide recommendations [13]. The National Comprehensive Cancer Network (NCCN), European consensus, and more recent Erasmus MC model refinement of these guidelines predict a 3-year metastatic risk between 0.21% (95% CI 0.10–0.31) for “low-risk” and up to 14% (95% CI 8.3–19.6) for “very high-risk” CSCC [8]. In cPOST, high-risk features summarised in Table 1 included extranodal extension in at least one node at least 20 mm in diameter, which was the most common criterion for trial inclusion in 50% of patients. These high-risk criteria were derived in part from a recursive partitioning analysis of the preceding negative POST trial which randomised CSCC patients to receive adjuvant radiation with or without concurrent carboplatin [14,15], as well as the available literature. We had assumed a 3-year disease-free survival of 55% in the placebo group, and a hazard ratio of 0.6. These criteria correctly identified a group with a high risk of recurrence or death despite receipt of both surgery and adjuvant radiation [3,14], with the 2-year DFS in the placebo arm of cPOST estimated at 64.1% (95% CI, 55.9 to 71.1). In contrast, the broader criteria used in the POST trial resulted in a 2-year DFS of 78% (95% CI, 72 to 85). In cPOST the benefit of adjuvant cemiplimab was observed in all subgroups of high-risk nodal and non-nodal disease, but its benefit beyond the trial-defined classification is unknown. In contrast to current guidelines, the cPOST criteria redefine and lay a novel foundation for the concept of “high-risk” disease and additionally predict for benefit of adjuvant cemiplimab. This highlights the urgent need for re-evaluation and harmonisation of staging systems and treatment guidelines.

The early splitting of the DFS curves at approximately 4 months in the cPOST trial demonstrates the impressive efficacy of cemiplimab to salvage patients with radiation-resistant disease following surgery, and highlights how quickly high-risk CSCC can relapse [3]. In the absence of prospective randomised trial data, there is considerable geographic variation in the use of adjuvant radiation [13,16,17]. While it is routinely offered to higher risk patients in the United States and Australia, this is not the case in some European countries. Current research efforts to quantify the benefit of adjuvant radiation are few beyond the “SCC-AFTER” phase 3 randomised control trial (ISRCTN54806122) [18] investigating the superiority of 45–60 Gy of adjuvant radiation within 4 months of surgery in resected BWH T2b/3 CSCC versus close observation alone. In the cPOST trial, all patients were required to receive a biologically equivalent dose of at least 50 Gy of radiation for eligibility, and the benefit of adjuvant cemiplimab beyond this context is unknown. While cPOST demonstrates that cemiplimab achieves a marked improvement in locoregional control (as well as a decrease in distant metastases), the trial does not address the role of radiation or provide justification for omission of radiation outside of a clinical trial.

With its crossover design allowing treatment with cemiplimab on relapse, cPOST has not demonstrated an overall survival benefit at the first analysis. There is an open question about the relative benefit of adjuvant cemiplimab versus treatment on relapse. It is noteworthy that the response rate to immunotherapy in the recurrent metastatic setting is ~50% with durable disease control, with the majority of these trial patients having had prior radiation [1,2,19]. In the adjuvant setting, cemiplimab achieves a 68% reduction in the risk of recurrence of CSCC after surgery and radiation therapy [3]. It is possible that not all patients in whom relapse will be prevented by adjuvant cemiplimab would be salvaged by waiting until relapse to treat with cemiplimab. Physicians should discuss both management options with patients to determine the best approach for an individual.

Another promising approach for locoregionally advanced CSCC is neoadjuvant immunotherapy. In phase 2 trials, impressive complete pathological response rates of 50–73% were obtained with up to four doses of neoadjuvant cemiplimab [20,21,22,23,24]. Given the shorter duration of immunotherapy albeit in a broader population, potential avoidance of adjuvant radiation therapy and related long-term toxicities, the durable control in patients who achieve a pathological complete response, and down-staging of disease opening the possibility of response adapted surgery, there is considerable enthusiasm and justification to investigate the benefits of neoadjuvant immunotherapy in phase 3 trials, such as NRG HN014 (NCT06568172) [25]. While neoadjuvant therapy has been shown to improve DFS compared to adjuvant therapy in stage III melanoma, the outstanding DFS achieved in cPOST will make it much more difficult to demonstrate a DFS difference [26].

The cPOST trial demonstrated that the receipt of adjuvant cemiplimab did not impair health-related QOL or functioning compared to placebo [3]. It is pertinent to note that despite the frequency of CSCC, there are no disease-specific QOL assessments that have been validated for patients with CSCC or “high-risk” CSCC. This is a key area for future research given the high proportion of cases in the elderly and the predilection of disease for sun-exposed sites such as the head and neck, which can impact a patient both functionally and aesthetically.

The cPOST trial has validated the high-risk eligibility criteria for resected locoregionally advanced CSCC and has defined adjuvant cemiplimab as a new standard of care option for patients with high-risk locoregionally advanced CSCC following post-operative radiation therapy.

## Figures and Tables

**Table 1:** cPOST high-risk criteria [3].

Extracapsular extension in ≥1 node at least 20 mm in diameter
Involvement of ≥3 nodes
In-transit metastases
T4 primary tumour
Radiologic/clinical involvement of named nerves
Local recurrence with at least one other adverse feature of ≥N2b, ≥T3 lesion, or poorly differentiated disease at least 20 mm in diameter
